# Neuromyelitis optica spectrum disorders and beyond: telling a story one hundred years later

**DOI:** 10.1055/s-0043-1770932

**Published:** 2023-06-28

**Authors:** Denis Bernardi Bichuetti

**Affiliations:** 1 Universidade Federal de São Paulo, Escola Paulista de Medicina, São Paulo SP, Brazil

To write about neuromyelitis optica spectrum disorders (NMOSD) is to tell a story. A story that starts in the late 19th century with the first case series assembled by Eugene Devic and his student, Fernand Gault 1 and fasts forwards one hundred years to 1999, with the establishment of the first set of diagnostic criteria, which detached NMO from a multiple sclerosis variant to set its own clinical grounds ( Figure 1 ).

**Figure:**
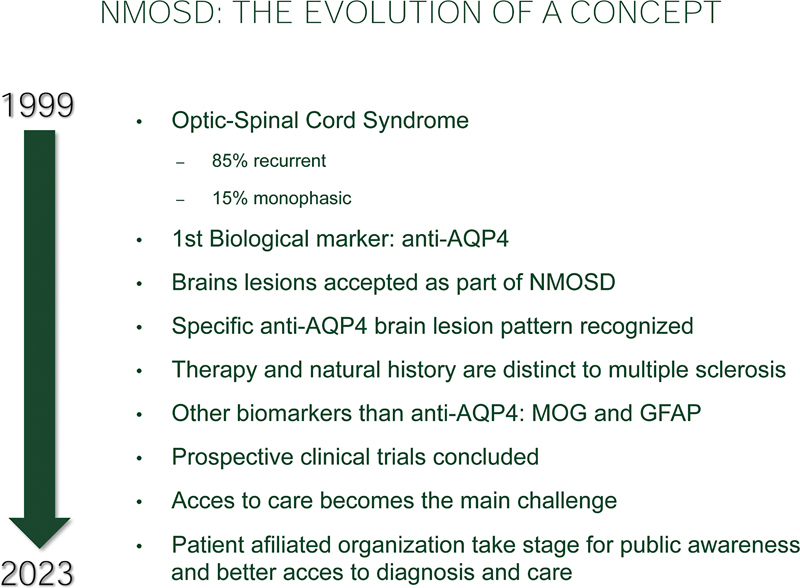
Figure 1

From that moment on, within ten years of research, it has been discovered and explained more about NMOSD pathophysiology and central nervous system (CNS) autoimmunity than in all years of demyelinating disease research so far. The narrative encompasses three sets of diagnostic criteria (1999, 2006, and 2015), at least two autoantibodies (anti-AQP4 and anti-MOG), four-phase three randomized controlled trials (eculizumab, inebilizumab, satralizumab and ravalizumab), an international collaborative foundation ( https://guthyjacksonfoundation.org ), national initiatives that joined patients and clinicians ( https://cdd.org.br and https://www.nmobrasil.com.br , to cite the larger two), and lots (really a lot) of observational, retrospective, and prospective data (countless references).

All along this story there are many Brazilian researchers, some that joined efforts in gathering observational clinical, epidemiological, and treatment analysis data, 2 3 4 5 6 7 8 taking active part in consensus diagnosis criteria 9 and even antibody discovery. 10 Not to emphasize (even more) our importance in this tale, there are many more physicians and researchers that contributes to this puzzle with increasing basic and clinical science research, and even more everyday caring for patients with NMOSD and Myelin oligodendrocyte glycoprotein antibody-associated disease (MOGAD) in the under resourced Brazilian Unified Health System ( *Sistema Único de Saúde* [SUS]) (impossible to cite all).

In the early ages of NMOSD, it was recognized only as an optico-spinal disease, but further along these years, the combined efforts of many disclosed that numerous patients suffer not only from brainstem syndromes, but diencephalic and encephalic inflammatory syndromes in distinct presentations. But… as things get more complex and we all get to know more about the pathophysiology of NMOSD, we are now aware that under this clinical umbrella there might actually be very distinct diseases, with different immunological mechanisms of central nervous system injury. At the moment, as I write this editorial, we are sentient of anti-AQP4 with its major complement and IL6 driven process, anti-MOG as an oligodendrocyte targeted inflammatory disease and anti-GFAP, thought with a very distinct clinical phenotype. Maybe in the future even more branches are added to this road.

In this issue of *Arquivos de Neuro-Psiquiatria* , Dr. Fragoso and colleagues 11 from Universidade de São Paulo and Pontifícia Universidade Católica do Rio Grande do Sul add some pages to this book by analyzing and categorizing imaging data from three sets of patients under this umbrella: those positive for anti-AQP4, positive for anti-MOG and double negative NMOSD.

Helping clinicians and patients that do not have access to antibody testing and relay only in clinical and imaging information to establish which syndrome is in front of them is of uttermost importance, especially for those practicing in underdeveloped or restricted resource settings, and for this reason we welcome the article from Dr. Fragoso and colleagues for establishing their differences and categorizing magnetic resonance imaging (MRI) data.

Still, it is worth emphasizing, as I recently heard in an international meeting, that “you do not have the final diagnosis until you earn it, and you earn it with the antibody”, especially when we are conscious that, based on the above-mentioned randomized controlled trials, some drugs might not work so well for anti-AQP4 negative subjects. So, having clinical and MRI helping us to establish which syndrome is in front us does not excuse us as physicians and medical society that we need to stress for universal access to antibody testing and better academic funding for immunological research that guide us on improved caring for all these patients. That shall be the next page to this story *, em alguns momentos escrita em português do Brasil!*

P.S.: I would like to extend apologies and acknowledge that I was not able to cite all Brazilian authors, physicians and organizations involved in research and care for patients with NMOSD. Everyone and every action counts.
